# A systematic review of developments in gripper technologies for rigid fabric parts

**DOI:** 10.1016/j.heliyon.2024.e40387

**Published:** 2024-11-19

**Authors:** Vivek Kumar, Paulo Jorge Coelho, Carlos Neves

**Affiliations:** a ESTG, Polytechnic University of Leiria, Leiria, 2411-901, Portugal; b Electrical Engineering Department, ESTG, Polytechnic University of Leiria, Leiria, 2411-901, Portugal; c Department of Mechanical Engineering, ESTG, Polytechnic University of Leiria, Leiria, 2411-901, Portugal; d Institute for Systems Engineering and Computers at Coimbra (Leiria Delegation), Polytechnic University of Leiria, Leiria, Portugal

**Keywords:** Systematic review, Robot grippers, Fabrics, Porous materials

## Abstract

This paper presents an inquiry of scholarly literature published in the last decade pertaining to the development of robot grippers for compressed fabric parts, which are both rigid and porous. The study is narrow and targeted. Previous literature reviews investigating technologies suitable for materials with similar properties were analysed, and the need for recent works addressing stiff and simultaneously permeable materials was identified. This work aspires to fill that gap with a systematic approach, for which the PRISMA reporting methodology is adopted. It entails scouting for publications with defined keywords, filtering based on predetermined constraints, and thoroughly examining the articles obtained from scientific databases. The study reveals that vacuum grippers are quite prevalent despite the inherent porosity of fibrous materials. The use of the Bernoulli and Coanda effects, and unconventional technologies like electro-adhesion are also gaining popularity. Intrusive instruments like needles are utilised regardless of their tendency to do surface damage. Moreover, hybrid grasping contraptions can be devised to overcome the limitations of their individual constituents. The operational efficiency of grippers can be further boosted with predictive modelling and sensors to execute a closed-loop system. Overall, the study conveys the latest advancements in multiple mechanisms available at designers' disposal, which can be implemented and optimised for the specific type of material, and its main technical contribution is in a specific gap in the literature by focusing on gripper technologies for handling rigid and porous fabric parts, which are commonly used in the automotive industry for acoustic applications.

## 1 Introduction

Unprecedented advancements in material science constantly pose new challenges to the automation industry, inducing progress in material handling capabilities. Complete automation in manufacturing, as opposed to manual labour that significantly impacts the efficiency of the production environments, has been pursued in the last decades. Although human-driven sub-processes are often slow, prone to error, and costlier, there are scenarios where it is the most viable solution [1]. Our general grippers, also known as robotic hands, can pick up various objects regardless of their properties. Therefore, tasks such as picking and placing new and/or specific materials, for which off-the-shelf automation solutions are not feasible, frequently get assigned to human operators.

**Figure 1:**
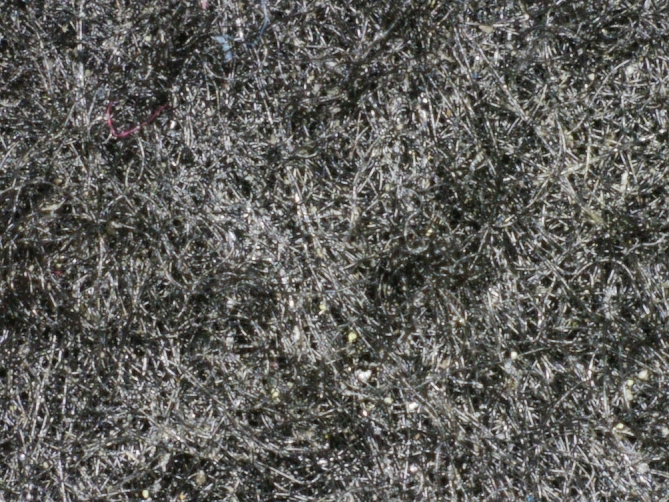
Surface texture of non-woven compressed polymer fabric material.

The target material for this survey is a specific semi-rigid part made of non-woven fabric material, which is frequently used in the automotive industry for acoustic applications. These parts are fairly strong, lightweight, and slightly elastic, and can have different 3-dimensional geometries. For its fabrication, individual polymer fibers are compressed and cut into specific shapes with a harder rim around the perimeter. Unlike textile sheets, these pads lack flaccidity and maintain their shape. They inherit the properties of a fibrous structure, namely porosity and an extremely rough surface with many loose fibers (see Fig. 1). One typical application of these parts includes placing them in a conventional injection mould cavity, where they are subjected to over-moulding. This ensures that the object is held firmly in the intended final shape. Prior to that, the geometry of individual parts is not consistent and sports wild curvatures, which further complicates the automation of this process.

Robot grippers (or end-effectors) are sometimes specialised for specific situations [2], and their study is an active area of research. The advent of various fiber-reinforced composites and the demand for automation in garment manufacturing are some of the related driving factors. Consequently, most of the available literature aims to manipulate thin sheets of flaccid fabric materials, as they are the most commonly used materials. Their limp nature presents unique challenges to the automation process but enables grabbing techniques like pinching or folding. However, these are not viable options in this study scenario due to the material's stiffness. Furthermore, the damage to the object and its surface must be minimised during the manipulation process, as is generally the case. These factors were considered to set the research parameters.

Several closely related review articles were sought after and then probed, but none of them investigated manipulation of rigid and simultaneously porous fabric objects. The aim to fill this gap was the motivation behind this research manuscript. A systematic approach was considered and the Preferred Reporting Items for Systematic Reviews and Meta-Analyses (PRISMA) [3] methodology was adopted. It prompts for an exhaustive search using precise keywords in various databases and search engines, followed by multiple elimination steps with well-defined criteria. Each step filters the search results in pursuit of the most pertinent publications. The articles obtained from this process were closely examined and classified based on the underlying principle of their design. Subsequently, inferences regarding applicable technologies and optimisation strategies were drawn and discussed in depth. The overall direction of advancements in appropriate technologies for the target application was thus outlined in this study.

The main contributions of this study are:

• Fills a significant gap in the existing literature by focusing on the automation of handling semi-rigid, porous materials used in industries like automotive manufacturing. Prior reviews have concentrated mainly on limp, textile-like materials, and this manuscript expands the scope to include materials with different properties, such as rigidity and porosity;
• Systematic identification and analysis of the recent technological advancements in robot grippers for handling rigid and porous fabric parts. It examines various mechanisms, providing a comprehensive overview of their capabilities, applications, and limitations;
• Highlights the potential of hybrid gripper technologies that combine multiple gripping principles to overcome individual limitations. It also emphasizes the integration of sensors within grippers to provide real-time feedback and improve control systems, which can significantly enhance the operational efficiency and adaptability of robotic systems in handling complex materials;
• Discusses existing technologies and their practical implications in industrial settings. It also suggests future research directions, including further testing in real-world environments, refining predictive models, and exploring new materials and innovative gripping mechanisms. This forward-looking perspective provides valuable guidance for ongoing and future research in robotic automation.

**Figure 2:**
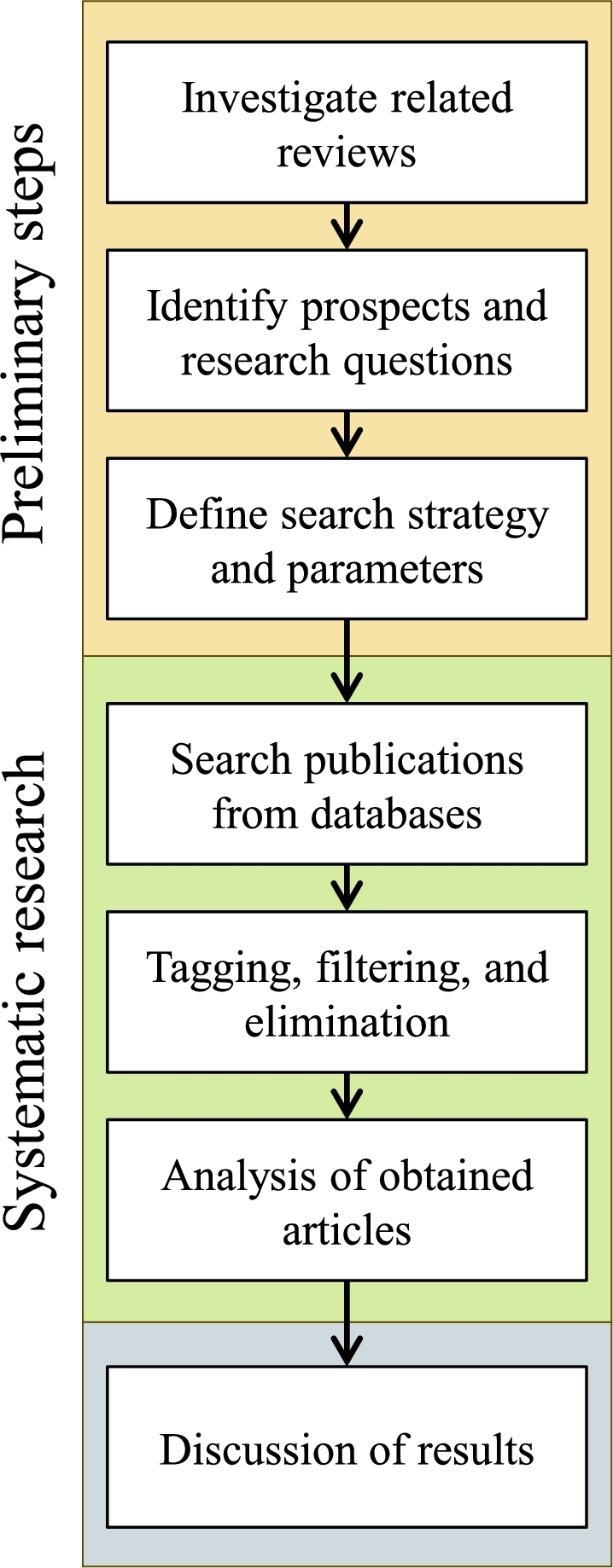
Flow of the systematic research and review process.

Fig. 2 illustrates the broad steps followed during the synthesis of this research, beginning with the identification and definitions of parameters, the search and filtration process, and the analysis of the results.

## 2 Related work

Like every other field of research, the sheer number of published scholarly works about automation has been on a steep rise in the last couple of decades. This trend has led to numerous distinct solutions. The design approach for grippers is diverse and often optimised for specific materials, objects, and/or environments [2]. Grippers designed to manipulate fabrics have attracted attention due to the ever-increasing demand. Consequently, multiple relevant research reviews have been made available over the years. Some of these are summarised in this section. These were selected based on the quality and extent of the research, target application(s), and the technologies investigated.

The recent study by Makris et al. [4] is a wide and in-depth bird's-eye view of all the technological aspects of the automatic assembly of non-rigid objects. They define non-rigid parts as objects with deformations larger than at least one of their dimensions. They have used a classification criterion described previously: one-dimensional objects like wires, two-dimensional materials like fabric sheets, and three-dimensional parts like human organs. Components that can fit multiple categories to some degree are also mentioned, along with the challenges and considerations for all types of objects. The authors provide extensive insights into simulation tactics as well. The overall focus is draping sheets on moulds while adapting their shape, which involves shape control, effects of grasping at various points, and effective tool dynamics. The use of sensors to optimise systems is explored, which includes identification, geometry definition, wrinkle or slack detection, and more. They conclude with further discussions of how all these factors affect control and other optimisation notes.

Wang et al. [5] have directed their focus on grasping techniques specifically for garment manufacturing. They have explored four technologies: pneumatic-based, electro-adhesive, intrusive, and simple mechanical grippers. Brief and concise explanations for these technologies have been provided as well. Many articles have identified that pneumatic technologies can be divided into vacuum suction cups, Coanda effect, and Bernoulli effect apparatuses. The authors describe their limited effectiveness with porous materials, and previous attempts towards applying these techniques. Applications of electrostatic adhesion like rolling, wrinkle-free manipulation, and ply separation have been described. Then, needles and brushes, i.e., intrusive mechanisms, are described, followed by hard and soft mechanical grippers. They wind up by asserting three distinct facets of their study: namely, the aspect of the gripper, aspects of fabrics, and aspects of the user.

In Borras et al. [6] the authors inspect various mechanical mechanisms used for fabric manipulation. They have compared general grippers with solutions targeting fabrics, and classified the solutions based on various parameters. One parameter is the number of “fingers”, namely single finger, two fingers, and multiple finger gripper. Then, the grippers are sorted depending on the usage of the fingers in various configurations, such as between fingers, fingers and a thumb, pinching, and so on. The motion dynamics have been described as well. A framework for defining the tasks involved in the process is their most remarkable contribution. They illustrate how the considered solutions manifest within their framework and how the framework can be used to guide the design of grippers for any system involving fabric manipulation.

Björnsson et al. [7] present an expansive overview of pick and place automation in the carbon fiber or glass fiber industry, and list articles that target dry fibers, prepregs, thermoplastics, and auxiliary materials. They have classified previous works based on addressed challenges like quality control, material, product or system level optimisation, and 2D or 3D material handling. Solutions based on vacuum, needles, mechanical clamping, and cryogenics have been identified. Furthermore, the authors note the effects of grip point distributions, and highlight active and passive solutions for dynamic reshaping of the material. The latter is required during the mould laying process, and includes both 2D and 3D shapes. Finally, various mechanisms adopted to optimise these processes are discussed.

A complete report is presented by Fantoni et al. in [2], wherein they have explored all aspects of grabbing involved in automation. Beginning with grasping and releasing principles as well as sensing and monitoring methods, they move on to detailed descriptions of applications in various scenarios. It includes general gripping, electronics assembly, micro-assembly, food industry, and logistics. The authors have explored cutting-edge developments in this field and some persisting problems. Many mention technologies applicable to fabrics and other porous objects throughout the document, including vacuum, needles, Velcro, Bernoulli, Coanda, electrostatic, mechanical, and cryogenic grippers. Multiple case studies for most mechanisms have also been meticulously documented and classified.

Finally, Koustoumpardis and Aspragathos [8] provides a compilation of the earliest descriptions of many technologies already discussed. They chose a classification based on broader working principles, namely, mechanical, pneumatic, adhesive electrostatic, and other surface attraction mechanisms. Moreover, the authors have provided brief explanations of the working, applications, and limitations of all the approaches. They concluded with a brief discussion of challenges at that time and how the engineering discipline would address them.

The examined reviews are listed in Table 1. Evidently, typical materials of interest are limp textiles, that are flaccid sheet-like objects which do not retain their shape. This is hardly surprising because fabrics are commonly used in the form of deformable sheets for garments or as engineering components. In addition, porous materials are hard to generalise. The solutions to automate the handling of porous materials depend massively on the implementation and the material itself. These factors contribute to a lack of suitable literature reviews for the specific application of this study. As described earlier, the target material is porous and rigid, and the process imposes certain restrictions as well. Although some of the methods mentioned in the previous reviews are applicable, many are not due to the difference in the properties of the target materials. Moreover, literature lacks a compiled list of technologies capable of handling such objects. There are recent developments in this aspect that are worth exploring as well. This article tries to addresses both of these facets.

**Table 1:** Previous review articles targeting similar materials.

Reviews	Title	Limp materials	Rigid materials	Mould placing
Makris et al. [4]	Automated assembly of non-rigid objects	✓		
Wang et al. [5]	Research progress of automatic grasping methods for garment fabrics	✓		
Borras et al. [6]	A Grasping-Centered Analysis for Cloth Manipulation	✓		
Björnsson et al. [7]	Automated material handling in composite manufacturing using pick-and-place systems – a review	✓		✓
Fantoni et al. [2]	Grasping devices and methods in automated production processes	✓		
Koustoumpardis & Aspragathos [8]	A Review of Gripping Devices for Fabric Handling	✓		
**This review**	A systematic review of developments in gripper technologies for rigid fabric parts		✓	✓

## 3 Research methodology

The Preferred Reporting Items for Systematic Reviews and Meta-Analyses (PRISMA) [3] strategy was adopted for the research. It entails an exhaustive search of scholarly works available on various databases with the chosen keywords and a publishing date range. Subsequently, articles are filtered out in steps according to several elimination criteria, and the remaining are retrieved and analysed. It should be noted that this systematic review is confined to only published scholarly works. This includes journal articles, conference papers, and book chapters but excludes patents, product reviews, and white papers.

Patents, in particular, are quite likely to describe commercially viable solutions, and the authors recognise that their removal might outcast some relevant information. Despite this, patents were considered not the most suitable sources because they can contain unstructured data or incomplete disclosures, making them challenging to analyse without specialized tools such as text mining and segmentation techniques. Their unstructured narratives, including titles, claims, and descriptions, add complexity to the analysis process [9]. Additionally, patents often lack peer-reviewed validation, depth of information, key information, and empirical data from test results typically required in academic analysis. Their focus on implementation and securing intellectual property, rather than presenting experimental findings, limits their relevance for theoretical developments. Moreover, patent analysis is often applied for strategic planning, technology forecasting, and competitor analysis—objectives that differ from the aims of this systematic literature review.

### 3.1 Research questions

The queries that this study pursues are as follows:

RQ1: What are the latest technological advancements in robot grippers for handling stiff and porous fabric parts?

RQ2: What strategies were adopted by previous works to optimise the gripping systems?

RQ3: How can sensors and implementing closed-loop systems improve efficiency?

RQ4: What are the limitations of the available techniques, and how can these be mitigated?

RQ5: Are system simulations a considerable option?

### 3.2 Search strategy

As previously established, the target material or the object can be categorised as a non-woven fabric pad. Preliminary searches with these keywords were limited, and broader keywords with fundamental material properties were defined to widen the scope of the search results. Hence, the keywords used in the search engines were “gripper for fabrics”, “gripper for textiles”, and “gripper for porous materials”. Moreover, to keep the review relevant and to limit the sheer number of search results, only articles published in the last decade (i.e., from January 2014 to April 2024) were considered.

Search engines and indexing services like IEEE Xplore, Scopus and ScienceDirect (by Elsevier), Springer Link, MDPI, and Google Scholar were used, along with lesser-known search engines like lens.org, semanticscholar.org, scite.ai, and consensus.app. The keywords were entered either as separate searches or as a boolean phrase, along with the date range.

### 3.3 Inclusion and exclusion criteria

The authors of this systematic review independently evaluated each potentially relevant paper to determine if it met the eligibility requirements. The studies were analysed, the gripper's technologies were classified, and the information was mapped to extract relevant information and connect it to industrial applications. The search was conducted on April 11, 2024 and updated on 11 October 2024. The following criteria were used to select studies for this systematic review: (1) studies about robot grippers with any of the above mentioned keywords; (2) studies that specifically targeted fabrics or porous materials; (3) studies which tested innovative designs or techniques and provide specific findings related to the tests; (4) technologies which can be used for stiff and porous materials; (5) original research studies published between 2014 and 2024; (6) works with valid DOIs; and (7) studies published in English.

The screening process was conducted in three steps, with distinct reasons for exclusion. The title, the abstracts, and conclusions were read to classify and tag all articles. The process of tagging eased the following filtering steps. As the first step, any documents unrelated to robot grippers were removed. This included other reviews and articles with missing information, regardless of the topic. At this stage, many articles describing technologies for actuators that can be used to design grippers were eliminated, along with papers that used grippers for other applications, particularly embedding sensors within grippers. The second elimination criteria employed segregation based on the application of the grippers. It was found that the majority of the short-listed articles described general grippers. Universal grippers are irreplaceable when the objects' type, material, shape, or weight can vary within the application. Despite that, they were neglected because the application is known and thus, the system can be optimised for the specific material. As such, only the articles that described systems for fabric or porous materials were selected for the next step. Moreover, some of the papers portrayed grippers designed for specific materials or objects (other than fabrics), which nullified their usability. Thus, the remaining papers about fabrics or porous materials were considered.

Finally, in the last filtering stage, the articles were subjected to a meticulous analysis to ensure their applicability for the targeted non-woven compressed fabric pads. This stage removed any techniques that exploited the ability of normal fabric sheets to be pinched, and mechanisms for draping sheets on moulds; which were the most common quirks of non-applicable solutions. The resulting papers were then sorted based on the working principle of the grippers and included for in-depth review, ensuring that only the most relevant and high-quality articles were selected. The above discussed criteria are summarised in Table 2 along with the respective reasoning and the stage at which it was deployed.

**Table 2:** Inclusion criteria for publications used at various stages.

Stage	Criteria	Details	Reason	Limitations
Identification	Keywords	Publications with matching search phrases in titles, abstracts and metadata	To find the most relevant articles available in literature	May exclude handful of relevant articles
	Publication date	Articles published within the last decade	Analyse only the latest advancements	Excludes technologies which haven't seen any development
Preliminary	Valid DOI	Scholarly works published in peer reviewed journals	To ensure the quality of the analysed articles	Excludes pre-prints and other media
	Duplicates	Publications with the same DOI	-	-
	Language	Primary language is English	To ease in retrieval and analysis	Excludes many on-topic texts
	Other reviews	Review articles with similar focus	To avoid redundancy	-
First round	Only grippers	Subjects pertaining to robot grippers	Narrow down the results	Excludes some usable technologies
Second round	Fabric grippers	The target material of study is fabric like or porous materials	Known material properties can be used for optimization	Excludes general or universal grippers
	Non-specialised grippers	Applications which are specialised for certain materials or conditions	To remove irrelevant technologies	Some mechanisms could be used after modifications
Third round	Applicability	Articles which can be used for the target non-woven, thick and stiff fabric materials	To remove irrelevant technologies	-

### 3.4 Extraction of information

The predominant classification used for the obtained articles is based on their technological characteristics, as presented in Table 4. The table also lists the type of publication, the primary target application or material of the study, their noteworthy contributions, and the limitations of the system. This information was meticulously extracted, analysed, and later used in the discussions. Moreover, if any sensor was used in the implementation, the study is also listed in Table 3. The authors grouped the articles depending on the measured parameters, namely, environmental factors like object detection, or internal variables like pressure. This is limited to sensors integrated within the gripping system, and does not include any external sensors that may have been used in the conducted tests. Likewise, articles that utilised simulations or mathematical modelling were also highlighted in the discussion section. Overall, the examination revolves around the applicability of the described technology concerning stiff porous fabric parts and improvements in the handling procedure.

**Table 3:** Analysed publications exploiting integrated sensors.

Sensors usage	Reviewed articles
Object (external) parameters	[10], [11], [12]
Internal parameters (for control)	[13], [14], [15], [16]

## 4 Results

**Figure 3:**
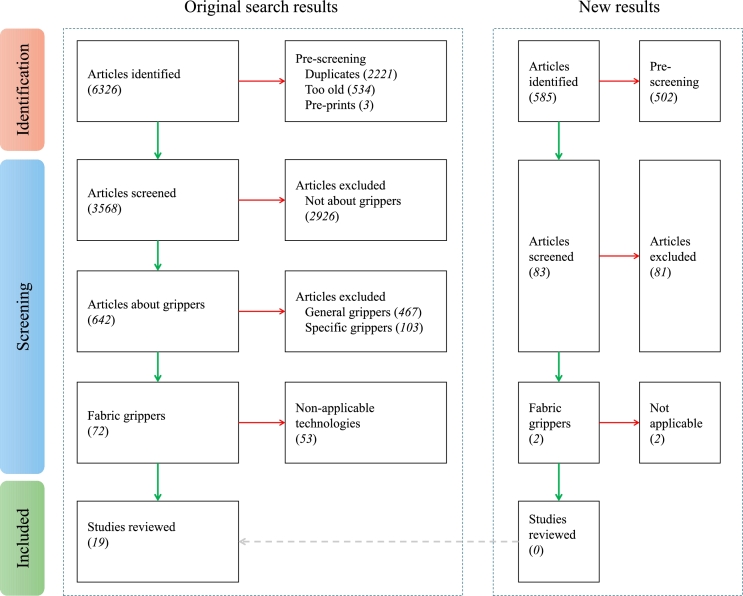
Flowchart of the selection of relevant scientific studies.

**Figure 4:**
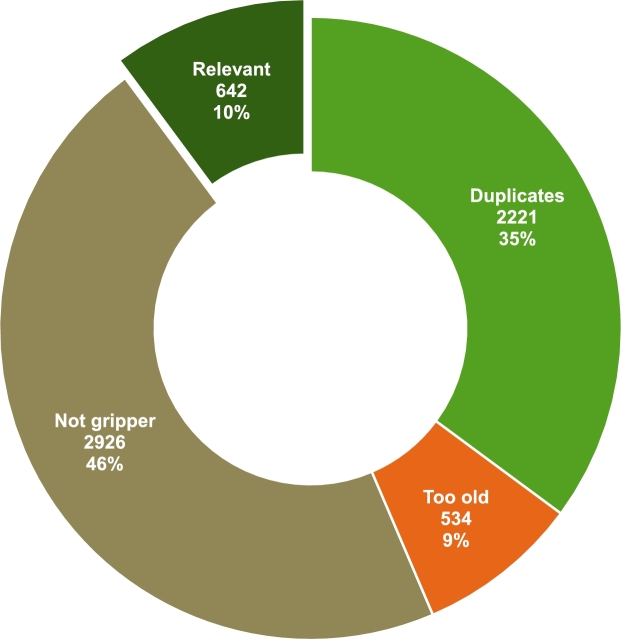
Scanning of search results in the first stage.

**Figure 5:**
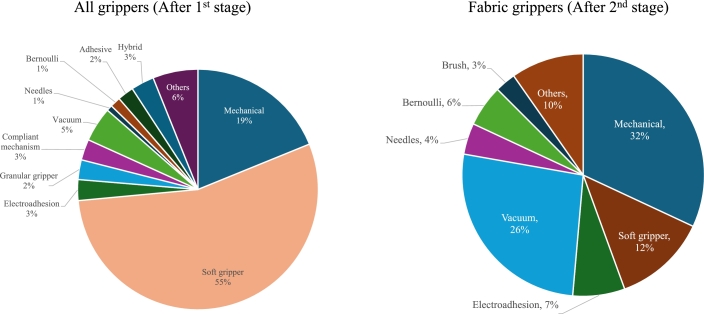
Distribution of technologies used in articles.

**Figure 6:**
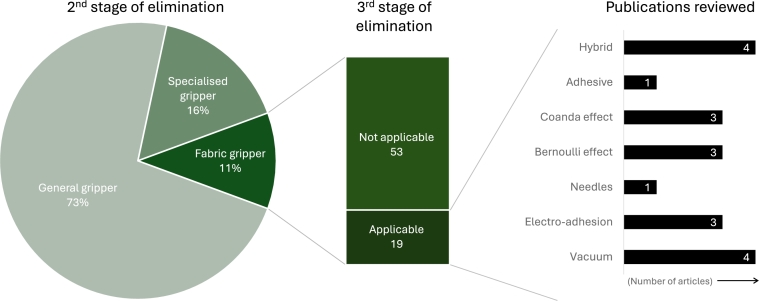
Filtering process used to obtain articles.

The search process returned a total of 6,326 results with the criteria established in Section 3, and had varying degrees of relevancy. Fig. 3 is an adapted version of the standard PRISMA flowchart, illustrating all the steps involved. The obtained results were exported and organised. Then, 2221 duplicates with the same Digital Object Identifiers (DOI), and 3 pre-prints were removed in a pre-screening phase, yielding 3568 documents for further screening. These papers underwent a title scanning or abstract examination stage, resulting in the elimination of 2,926 articles. Distributions from this first round of scanning are illustrated in Fig. 4. The 642 articles describing grippers were passed on for the next stage of filtering. Out of these, 467 studies (73%) were related to general application grippers, and 103 manuscripts (16%) were about specialised grippers unsuitable for the task. From the remaining 72 studies, 53 were also removed due to their applicability to only flexible fabric objects. The assortment of technologies and mechanisms used by the authors that were considered at various stages of the filtering process are presented in Fig. 5 and Fig. 6. Even though a handful of them were not directly addressing similar stiff materials, the technology would work for the target application. Finally, the quantitative synthesis and meta-analysis incorporated the remaining 19 studies. Table 4 lists all the articles and their underlying technology, arranged in descending order of date of publication. The working principle is also used to club the articles together in the following summaries. Other than the major groups, there were solutions that applied two or more mechanisms to achieve their gripping goals, and are categorised as “hybrids”.

**Table 4:** Analysis of the articles included in the study.

Ref.	Year	Title	Type	Technology	Application	Distinct features	Limitations
[13]	2024	Multifunctional Soft Gripper With Microneedles and Integrated Sensing for Robotic Fabric Handling	Journal Article	Hybrid (needles and vacuum)	Picking up different types of fabrics from stacks	Retractable micro needles, compliant structure to avoid damage, pressure sensor for vacuum	Might only apply to flexible or pinchable materials
[14]	2023	Automated Stack Singulation for Technical Textiles Using Sensor Supervised Low Pressure Suction Grippers	Conference Paper	Coanda effect	Manipulating single sheets of carbon fiber textile	Robust mathematical model backed by simulations and tests, distributed air-flow channels	Low effectiveness at ply singulation from a stack of sheets
[10]	2023	Mechatronic Design of Two-Jaw Pleating End-Effector for Large-Scale Carbon Fibre Reinforced Polymer Manufacturing	Journal Article	Vacuum	Moving pleats of nylon sheets onto CFRP moulds	Integrated sensor for enhanced precision and accuracy	Testing done in highly controlled environments
[17]	2023	Gripper for Manipulating Empty Bag Sacks	Conference Paper	Needles	Handling sacks of multiple materials for packaging	Investigation of the effect of varying the angle and pressure of actuators	Applicable to large parts, frequently required maintenance
[18]	2023	Finite element modelling of grasping porous materials in robotics cells	Journal Article	Bernoulli	Flexible textiles with varying surface roughness	Vibration absorbing ring to stabilise the system, FEM analysis of the grasping dynamics	Model could be considered too simplified and theoretical
[19]	2023	Gripping Device for Textile Materials	Journal Article	Bernoulli	Gripping sheets with unstable orientations	Extensive mathematical modelling accounting for many variables, power analysis	System is susceptible to vibrational disturbances
[20]	2022	Vacuum System for Reinforcing Fabric Handling	Journal Article	Vacuum	Automatic lay-up in composite manufacturing	Low pressure, high flow, simple and commonly available vacuum generator	Influence of permeability is not well established
[21]	2022	Robotic Fabric Fusing using a Novel Electroadhesion Gripper	Conference Paper	Electro-adhesion	Picking and aligning cloth pieces automatically	Simple yet effective actuator design, vision based sensing for alignment	Not scalable to small parts, vulnerable to environmental factors
[22]	2022	Analysis of grasping deformation of textile fabric based on fluid structure coupling	Journal Article	Bernoulli	Modelling behaviour of fabric sheets while gripping	Computational analysis of design parameters and tests for optimization	Does not account for material properties like porosity
[23]	2021	Dexterous textile manipulation using electroadhesive fingers	Conference Paper	Electro-adhesion	Textile manipulation in different condition	Employs electro-adhesion for ply separation and a mechanical finger for a sturdy grip, tests on materials with different textures	Constricted to edges and corners to achieve a grip
[24]	2021	Universal gripper for fabrics – design, validation and integration	Journal Article	Hybrid (vacuum, intrusion and pinching)	Universal fabric gripper for automatic handling	Multi-instrument gripper for various scenarios, and a minimalistic selection flow for them	Material specificity, and need for precise pressure control
[25]	2019	Study of Grippers in Automatic Handling of Nonwoven Material	Journal Article	Vacuum	Effectiveness of vacuum cups for non-woven fabrics	Vigorous tests of different vacuum cups for a vast variety of non-woven fabric sheets	Results possibly valid for a narrow category of materials
[26]	2019	A New Electrostatic Gripper for Flexible Handling of Fabrics in Automated Garment Manufacturing	Conference Paper	Electro-adhesion	Manipulating textiles while flattening wrinkles	Innovative electrode design and a secondary mechanism to straighten the grabbed sheet, expansive tests on different type of fabrics	May not be compatible with thick and heavy fabrics
[15]	2018	Development of a Handling System with Integrated Sensors for Textile Preforms using Additive Manufacturing	Journal Article	Vacuum	Handling fabric components for reinforced composites	Simple and elegant actuator design with an integrated pressure sensor, precise testing	Possibly limited to specific manufacturing settings
[16]	2018	Tack and deformation based sensorised gripping using conductive hot melt adhesive	Conference Paper	Adhesive	General gripper with minimal complexity	Novel design which exploits temperature dependant tackiness of thermoplastics	Unreliable attraction force needs to be rectified
[27]	2017	Automated Stamp Forming of Continuous Fiber Reinforced Thermoplastics for Complex Shell Geometries	Journal Article	Hybrid (vacuum and needles)	Automating the stamp forming process	In-depth analysis of wrinkles and corner deformation after laying up the fabric	Very specific application


Ref.	Year	Title	Type	Technology	Application	Distinct features	Limitations
[28]	2016	A self-aligning gripper using an electrostatic/gecko-like adhesive	Conference Paper	Hybrid (electrostatic and dry-adhesion)	Efficient and dependable handling of flat objects	A multimodal gripper with a new electro-adhesion electrode design and a gecko-like surface for enhanced shear grip	Debris or dust hampers performance and needs to be mitigated
[11]	2016	Sustainable Manufacturing Through Energy Efficient Handling Processes	Journal Article	Coanda effect	Analysis and optimization for sustainability	Analysis of various factors, and use of sensors to optimize operational energy efficiency	High initial set-up cost and calibration required
[12]	2014	Intelligent gripper technology for the handling of carbon fiber	Journal Article	Coanda effect	Efficient gripper for carbon fiber textiles	Optimized gripper based on Coanda effect with an in-built sensor and distinct air channels with precise control	Sensing element is inherently limited to conductive materials

Furthermore, an updated search and selection process was performed using the above described process during the review of this article. The date of publication range was extended to include articles published until October 2024, while other parameters and criteria were kept constant. This yielded 585 articles, from which 2 remained till the final round, wherein they were also removed because of their non-applicability. This sub-process is marked within new results in Fig. 3. Still, the data obtained was not used to update any other diagrams in the manuscript, reflecting only data from the original search results.

The following sections organise the results by several types of technologies, namely pneumatic (subsection 4.1), Electro-adhesion based (subsection 4.2), intrusive needles (subsection 4.3), adhesives (subsection 4.4) and hybrids (subsection 4.5).

### 4.1 Pneumatic

#### 4.1.1 Vacuum

Chen et al. [10] presented the Vacuum Infusion Moulding End-Effector (VIMEE), a mechatronic tool designed to automate pleating in large-scale Carbon Fibre Reinforced Polymers (CFRP) manufacturing. It aims to reduce manual labour and improve efficiency by integrating components for pleat loading, height detection, and migration. The authors have integrated sensors and control systems to make the system more robust. Testing in a simulated vacuum bagging process showed its effectiveness in manipulating nylon pleats. However, the VIMEE prototype has only been tested in a controlled environment, suggesting further testing in actual manufacturing settings is necessary. If successful, VIMEE could significantly reduce labour costs and improve efficiency.

Kužel and Růžek [20] proposed a vacuum grabbing mechanism developed to handle dry reinforcing fabrics without damaging them, primarily for the aerospace industry. The device combines a custom-made suction grid attached to an industrial vacuum cleaner with variable power settings. The system's efficiency was confirmed through rigorous testing on various fabric types, proving its ability to manage diverse weaves and weights without structural damage. However, the load capacity of the vacuum system is influenced by fabric porosity and does not expand linearly with suction power. The technology offers flexibility, reduced material damage risk, and higher precision in fabric manipulation, suggesting potential cost savings and efficiency gains in composite manufacturing.

Cubric and Cubric [25] presented a study that addresses the effectiveness of vacuum grippers in handling non-woven materials. The test apparatus included a compressor, air preparation unit, and various vacuum suction caps. It was used to assess the gripping ability of 14 non-woven materials, which were different blends of polypropylene, polyester, and viscose fibers. The grippers were tested under varying inlet pressures (2 to 5 bar). The results showed that the flat suction cap performed well, especially when used with the L-type ejector and positioned in the middle of the material. The tiny suction cap showed limited efficacy, with improved outcomes near the material's edge under high pressures. Large suction cups were not effective at all. The study suggests that while vacuum grippers have the potential for automated handling of deform-able fabrics, their effectiveness depends on specific variables such as suction cap type, gripper positioning, and pressure settings.

Brink et al. [15] develop a simple yet effective automated handling system with sensors for managing textile preforms, specifically for fiber-reinforced plastic components. It comprises a fan mounted on a housing with a front grill made by fuse deposition modelling (FDM). The fan generates the desired lifting force, and the strain gauges accurately measure the bending of the grill to determine the gripping force applied. The system could dynamically adjust the gripping force based on the number of manipulated layers, ensuring precise control without damaging the materials. The system successfully enhanced the precision and efficiency of textile handling processes, which are crucial for composite material production. However, the system's application may be limited to specific environments and materials, necessitating further adaptations for broader use.

#### 4.1.2 Bernoulli effect

Mykhailyshyn et al. [19] developed an improved Bernoulli gripping device with an anti-vibration insert for handling textile materials in industrial processes. Like other Bernoulli's effect-based actuators, their device uses pressurised air but is optimised to handle non-rigid and porous items. The design addresses issues like material deformation and ineffective grasping. The authors have deduced a robust mathematical model to maximise the attraction force by tweaking various parameters based on the mass and other properties of the material. Analysis of the material includes using a 3D scanning profilometer to gauge the surface texture. The prowess of the model is further supported by a Finite Element Model (FEM) analysis presented in a previous study [18]. Equipped with this, the authors simulated the effects of air channel geometry, inlet pressure, and pressure distribution. The resultant device shows significant improvements in energy economy and operational versatility. The most significant contribution is a 3D-printed anti-vibration insert, which provides mechanical stability. The ability to adjust compressed air flow during operation is also exploited to attain a firm grip. The authors prescribe further optimisation in finite element analysis to address residual vibrations and methods to adapt to variable textile characteristics.

Zhang et al. [22] investigated deformation issues during automated transfer processes in the garment industry. The research employed multiple non-contact Bernoulli suction cups mounted on spring buffer rods to optimise adsorption force to reduce fabric deformation. A fluid-structure coupling adsorption model was established as well. Through simulations and experiments, the adsorption force was computed under various suction cup configurations and inlet pressures. This included the number of cups, their relative distances, and the air pressure. The study found that a setup with five Bernoulli suction cups with an inlet pressure of 0.3 MPa resulted in the least deformation. By changing various parameters, the authors could boost the automation capabilities while preserving fabric quality. However, the study acknowledges limitations, such as omitting fabric air permeability in simulations.

#### 4.1.3 Coanda effect

Wirth et al. [14] developed an automated system for separating single fabric plies from fabric stacks, a challenge in handling air-permeable technical textiles. The system used low-pressure suction grippers with differential pressure sensors to overcome the flexible and porous nature of the material. The noteworthy contribution is the predictive modelling of suction pressure based on many factors like material properties, tool geometry, vacuum generation, and load. This, along with sensor feedback, is used to regulate the suction process effectively. The equipment setup includes perforated suction plates and a vacuum-generating Coandă ejector. The system achieved a success rate of over 99% in separating single layers from fabric stacks, demonstrating potential for practical application in industrial settings like fiber-reinforced plastics production for automotive and aerospace components. However, the study highlighted limitations, such as handling multiple layers simultaneously and the need for extensive experimental data for predictive modelling.

Fleischer et al. [12] aimed to develop an unprecedented low-pressure gripper system using the Coanda Effect to automate the handling of carbon fiber textiles. The technology aimed to reduce manual labour intensity and high production costs in manufacturing Carbon Fiber-Reinforced Polymers (CFRP). They performed a cause analysis for the common problems that occur specifically while handling textile materials with pneumatic systems. It suggested that low energy efficiency is the major drawback of such systems, as well as lack of dependability and flexibility. This gap is prominent when removing pieces from a stack, handling a thin singular piece, handling multi-layered objects, and more. Their solution to these issues is in the form of a gripper with several air inlet holes with an in-built sensor. The sensors are constructed in the form of two concentric electrodes, which are carefully placed along the holes. The conductivity of carbon fibers enables the measurement of contact pressure and pressure differential, which is used to control the suction pressure. This enhances the energy efficiency, reliability, and adaptability of the system. Test results showed a significant reduction in compressed air consumption by up to 75%, which directly translates to lower operational costs. Moreover, the study also implemented a decentralised control system for regulating suction power, allowing real-time adjustments based on gripping needs. Further analysis presented in [11] revealed that real-time monitoring and dynamic suction power adjustment resulted in over 50% energy savings. Though the system may be limited to specific settings and material types, and the installation process could incur higher initial costs as well. The benefits, particularly in terms of energy savings and potential cost reductions in long-term operations, highlight the value of this innovative approach in sustainable manufacturing practices.

### 4.2 Electro-adhesion

He et al. [21] have developed a robotic system revolving around electro-adhesion phenomena, to automate the fabric fusing process in garment manufacturing. The system, which combines an electroadhesion gripper with actuated pins and camera-based sensing, ensures wrinkle-free fabric alignment, which humans typically handle manually. The system's effectiveness was demonstrated in controlled laboratory conditions and on actual garment manufacturing floors, with over 80% success rate in picking up fabric pieces under specific humidity conditions. The gripper's functioning is highly regulated by voltage changes based on fabric type and environmental conditions, particularly humidity. Despite its limitations, the system's benefits, such as reduced labour intensity, better fabric handling uniformity, and the ability to operate with varied materials, outweigh its constraints. The system's adaptability to fabric weights and types without damage further highlights its potential to revolutionise fabric handling procedures in the garment sector.

Digumarti et al. [23] presented a two-fingered gripper with an electro-adhesive skin to automate textile manipulation in garment manufacturing. The technology targets small places to separate fabric plies without harming them, overcoming limitations caused by the delicate nature of materials. The gripper attaches to fabric corners with one hard finger, lifting fabric corners, while the other movable finger helps grab and manipulate the material. The gripper demonstrated high precision and low damage in handling various materials, forms, and sizes. It successfully folded and repositioned smoother surfaces, while rough textures had issues. The gripper's flexibility to flat and non-flat materials improved its utility in real-world industrial applications. However, the study noted limitations such as reduced effectiveness on rougher surfaces and environmental conditions, which could impair performance. Overall, the development of this technology represents a significant advancement in textile industry automation, promising better efficiency and reduced dependency on manual labour.

The study by Sun and Zhang [26] presents a unique electrostatic gripper designed for automated garment manufacturing that is capable of handling various materials without harm. The gripper consists of four adjustable electro-adhesion pads, a slider-crank mechanism for expanding or contracting pads, a servo motor for actuation, and an ambient light sensor for fabric grabbing success. Experiments show the gripper can handle 30 types of fabrics, with the saw-tooth electrode design significantly increasing adhesion force. The gripper's adaptability to diverse fabric types and ability to flatten materials without harm are significant breakthroughs in textile automation. However, limitations like handling thick or heavy materials and susceptibility to environmental pollutants like dust highlight the potential for future improvements. The study suggests the gripper could replace manual handling operations in garment manufacturing, enhancing production efficiency and reducing human resources reliance.

### 4.3 Needles

Rizescu and Rizescu [17] developed a pneumatic gripper to automate the handling and palletising of empty bag sacks. The gripper uses thin needles to penetrate and lift materials like cardboard, polyurethane foam, expanded polystyrene, sackcloth, and raffia. The device's design includes cone-shaped needles, pneumatic cylinders, and helical compression springs, optimising energy consumption and performance. The authors analysed the effects of different attack angles and applied pressures for effective grasping and positioning. Experimental results closely matched theoretical models, demonstrating the gripper's reliability and efficiency. Although it requires periodic maintenance, the gripper significantly reduces manual labour and improves safety in packaging processes.

### 4.4 Adhesives

Hughes and Iida [16] developed a unique gripping mechanism using a Conductive Hot Melt Adhesive (CHMA). It constitutes of conductive carbon black particles integrated within a layer of thermoplastic and a temperature control system. The mechanism aims to provide a universal solution for grasping various objects by adjusting the adhesive's tackiness and deformation. The system adjusts its properties to efficiently handle objects of different shapes, sizes, and materials; which includes roughly textured textiles. The CHMA-based gripper demonstrated its flexibility owing to its inherent pressure-sensing capabilities. The study highlighted its benefits, such as its ability to handle various materials, simplified system design, and improved utility in industrial settings. However, it also noted variability in tack force measurements, which could affect the system's reliability and consistency. Further work is suggested to refine the material's properties and control algorithms for a more robust and predictable system.

### 4.5 Hybrids

Ku et al. [13] present a versatile soft robotic gripper designed to manipulate different types of fabrics in the garment manufacturing sector. The gripper, equipped with micro-needles and integrated air pressure sensors, utilises dual actuation modes. The two mechanisms - pinching and suction, can be switched swiftly based on the air permeability of the fabric, which is measured initially via a sensor. A compliant mechanism enables adaptive handling methods as well as reduces harm to fabrics. The gripper displayed dependability and efficiency, with 95% and 100% success rates in separating air-permeable and non-air-permeable textiles throughout 10,000 actuation cycles. While the gripper is efficient, it has difficulties dealing with extremely thin or highly flexible textiles, which indicates a requirement for more improvement in its operations. Although there are some restrictions, the gripper's novel design represents noteworthy progress in automated garment production, offering significant enhancements in handling efficiency and material safety.

Ebraheem et al. [24] developed a universal gripper that can handle various textiles without causing deformation or damage. The gripper combines vacuum, intrusion, and pinching methods, which were tested and validated using a robot arm. The researchers tested many parameters to optimise them based on the material properties. Their examination included static tests, dynamic tests, and ply separation tasks. The vacuum technology was effective for impermeable materials, intrusion for porous textiles, and pinch technology for flexible fabrics. The authors also developed a simple algorithm to determine the most effective technique for a given material. The system requires precise pressure adjustments for optimal performance and has limitations based on material properties, which can be considered a liability. However, the gripper presents significant advancement in versatility and integrates seamlessly with existing robotic systems.

Behrens et al. [27] presented a fully automated process for forming complex shell geometries of continuous fiber-reinforced thermoplastics, specifically for applications like battery trays for plug-in hybrid vehicles. The authors developed a multi-material gripper system using vacuum suction and needles equipped with an infrared radiator. The emphasis was on minimal defects during the draping process, and successful experiments were conducted and analysed. The study highlights the importance of precise temperature control and handling to prevent wrinkling and fiber fractures. It was concluded that automated stamp forming is viable for producing complex FRP parts at scale, particularly in automotive manufacturing. However, challenges such as handling hot, limp materials and precise temperature management were identified. These insights contribute to the large-scale application of fiber-reinforced thermoplastics in industries with critical lightweight and structurally sound components.

Another innovative design is described by Dadkhah et al. in [29], where they have developed a complete gripper system for a wide variety of flat materials. Their application aims to improve automation and decrease reliance on vacuum technology as well as manual tasks. The design consists of sub-divided flexible electroadhesive electrodes mounted on an actuator that can pull and release them onto the object's surface. The electrode was fabricated as non-coplanar copper traces on a polyimide substrate. Their key innovation is the introduction of a Gecko-like dry adhesive surface on the electro-adhesive plate, composed of sub-millimeter triangular wedges. This substantially increases the grasping shear force, as is evident from their test results, which they performed on diverse materials and surface textures. This included a few different types of fabric sheets, all of which experienced extremely low force compared to other materials. Their future prospects included a more robust design with fewer moving parts and further testing in industrial settings.

## 5 Discussion

### 5.1 Interpretation

This article has thoroughly examined the usage of technological advancements in robot grippers for handling stiff and porous fabric parts. The second stage of filtering, described in subsection 3.3, excluded multi-purpose grippers, although some of them can be optimised for the target material. Additionally, needles or pin penetration-based technologies, for example, have become industry standards and might fail to inspire new academic research. Some technologies like hydro-adhesion and others described in previous reviews did not appear in the filtered search results, though they will be explored in subsection 5.2. Various inferences drawn through rigorous analysis of the selected articles are discussed below.

Vacuum or other negative pressure-based grippers are prevalent, despite the porosity of the materials. Various strategies have been adopted to circumnavigate the limitations of traditional vacuum grippers. One of the most common approaches involves deploying sensors to measure several parameters in real-time. Measuring the changes in pressure generated during the operation is especially advantageous [15], [12]. The data can infer the success or failure at the picking stage or determine if multiple objects have been picked up. Modulation and optimisation of the vacuum pressure can be done as well. For porous materials, the gripper can have distributed channels, which avoids the loss of vacuum pressure, thus increasing the efficiency and effectiveness of the grip [14], [18]. These can be controlled as well to reduce leakage where no contact with the material is made [12]. Larger vacuum cups are often less effective than smaller ones, but their performance varies with surface texture for both. Rougher textures generally yield lower lifting force [25]. Furthermore, the combination of low pressure and high flow rate effectively handles porous materials [20], [15]. However, it poses its own challenges, like harder control and a predisposition to pick up multiple objects from a stack.

Other pneumatic mechanisms, chiefly grippers based on the Bernoulli and Coanda effects, are also available. The Bernoulli effect, in particular, is a widely available technology used across many industries. Contactless grip is a unique advantage of this approach, which can be considered if damage to the object's surface must be minimised [22]. The downside of Bernoulli-based grippers is their relatively low reliability and lack of orientation control. Both can be attenuated by utilising sensors or secondary mechanisms [12]. Furthermore, simulation and mathematical modelling are commonly deployed to optimise various parameters for a given set of material properties like porosity, mass, and surface texture [14], [19], [18], [22]. A more precise control over the generated force can be achieved by modulating the air flow rate.

Electro-adhesion has proven to be remarkably successful for fibrous materials. A major advantage of this technology is the ability to exert force over a large area, which makes it perfect for handling large sheets of material [21]. Textiles tend to have extremely rough and uneven surfaces, which can differ widely between types of fabrics. This translates to a large variation in the obtained adhesive pressure [21], [23], [26], and can be difficult to predict by theoretical calculations. Due to the exponential decay of the force further away from the actuator, electro-adhesive grippers excel in ply separation tasks, picking up only the topmost sheet from a stack. The most popular fabrication techniques for the electrodes are simple PCBs [21] and flexible PCBs [26], followed by silicon moulding [23]. Additionally, the shape of the electrode can have a massive influence on the electric field [26] and hence on the force obtained. Moreover, modifications to the electro-adhesive surface can overcome the effect of diminished surface contact. This can be in the form of flexible or stretchable electrodes, and/or dry-adhesive or Gecko-like surfaces [29].

Intrusive mechanisms like needles and brushes are relatively popular, with abundant commercial availability. Although surface damage is an inherent concern with such techniques, they do offer many advantages. The grip strength and reliability are superior to other methods and present small variations in different materials. They also excels in ply separation tasks, as the depth of partial penetration can be controlled. The angle of penetration plays a prominent part in the achieved grip strength [17]. The damage caused can be mitigated by using thinner micro needles [13], which are gaining momentum in the industry. Likewise, brushes or Velcro-like stiff strands are another class of intrusive grasping technology. Their utility is partly due to their affinity for fabric-like materials and relatively lower power consumption. However, surface detrition is significant, chiefly while the object is being released.

Adhesion or surface attraction through other means is yet another method available for implementation. Although chemical adhesives can be used for this purpose, these raise concerns regarding residues, surface deterioration, and inconsistent forces. The solutions to these issues can be in the form of dry adhesion and active adhesion. Dry adhesion employs Van Der Wall attraction force by radically increasing the effective contact surface area, similar to Geckos' feet. This is often used with other mechanisms as it works extremely well for shear forces but cannot generate a normal force. On the other hand, active adhesion has a surface with a switchable attraction mechanism. There are many ways of realising this technology, which mitigates most of the aforementioned issues [16]. It is a relatively new mechanism enabled by recent progress in material science and composite materials. It should be noted that hydro-adhesion, which was developed decades ago, can also fall under this category.

Hybrid technologies combine more than one working principle and have witnessed great advancements. Mechanisms are combined to complement each other's limitations, thus yielding a more robust system. Vacuum, for example, has very limited efficacy with porous materials but can be combined with intrusive mechanisms. Contraptions like this can be used for larger sheets to keep the material wrinkle-free, and the edges planar [24]. Integrating a soft body or a compliant mechanism reduces the risk of damaging objects during the gripping process [13]. Soft grippers can specifically feature micro needles that just slightly penetrate the object's surface, hence reducing the damage. Furthermore, combining various mechanisms to make the end-effectors more adaptable and multi-purpose is a frequently occurring approach [29]. This is a desirable trait, particularly when the material to be handled is unknown or there are other practical constraints.

### 5.2 Other technologies

Due to the time frame used in the inclusion criteria (10 years), the search somehow limited the research to only recent developments, and some noteworthy techniques were not included. Here is a description of some of those technologies described in articles that were not found through the systematic approach. Nevertheless, the authors considered them important in improving this discussion.

#### 5.2.1 Cryogenic (hydro-adhesive) grippers

This unique technology operates by freezing liquid to achieve prehension. The object's surface is drizzled with water, which freezes when it comes in contact with a pre-cooled surface. The fibres stuck in the ice act as the adhesive media. One of the earliest prototypes, by Günther et al. [30], used Peltier heat pump modules and attained a firm grip within a second. The type of material has a negligible effect on the adhesive force and is mildly affected by the surface ‘fluffiness’. Once frozen, the mechanism provides substantial adhesive force and prevents any slippage during robot movements. Significant challenges are involved in implementing this mechanism, like the risk of contamination and the requirement of a relatively flat surface. Limited commercially available cryo-grippers are used in the industry, some of which are specifically designed for fabrics. Their utility was demonstrated by Tarsha et al. [31] to design their multi-point fabric draping end effector.

#### 5.2.2 Soft grippers

Although it was mentioned only briefly in the previous sections, soft gripper and soft robotics, in general, have witnessed a substantial amount of development in recent years. Soft grippers refer to a general category of compliant mechanisms. They are flexible, both literally and regarding adaptability towards the environment. There is a litany of ways this can be implemented, with varying degree of compliance and degrees of freedom [28] and using pneumatic actuators, cable control, shape memory materials, or active polymers. It is common to see alterations to the surface of the soft grippers, which can be in the form of micro-needles, electro-adhesion, or dry adhesion. These combinations are developed to specialise in a particular material. Since this technology is recent, many challenges are yet to be resolved, such as the lack of precision and repeatability, and low grasping forces. The system's accuracy can be expanded using sensors, as with any other mechanism.

#### 5.2.3 Sensors

Sensor integration within the end-effector is a prevailing practice. Progresses included detecting the object itself, its orientation, surface features, porosity, presence of wrinkles or deformities, changes in pressure, and other inputs. A closed loop system renders a significantly more reliable system, as is evident from numerous examples [13], [15]. The data can be used to adjust control variables in real time or improve prediction models and simulations. The latter can provide vital insights into the operation of the gripper. Material response to the gripping mechanism is the most prevalent approach, often using some form of Finite Elements Models (FEM) [18], [14], [15], [12]. Pneumatic-based technologies benefit from simulation models for developing control strategies, though they often use fluid dynamics simulation methods [22]. Prudent analysis of predictive models is used to aid the gripper design process, as well as to set and optimize control parameters.

#### 5.2.4 Using additive manufacturing

Additive manufacturing, especially 3D printed fabrication of robotic grippers can provide a versatile and efficient way to address challenges like shape compliance, structural rigidity, weight, and sensor integration. Through advanced multi-material 3D printing, researchers have developed grippers that seamlessly combine soft, rigid, and conductive materials—such as polymers, polymer nanocomposites, and metal—into a single structure with minimal assembly required [32]. This approach enables the creation of multi-functional grippers that can adapt their stiffness and shape to handle a wide variety of objects, including fragile, irregular, or flexible items like fabrics. For example, 3D-printed grippers with tunable stiffness, achieved through Joule heating of conductive polylactic acid, can conform to the shape of different objects while maintaining the necessary rigidity for effective handling [33]. Additionally, embedded sensors, such as those printed using thermoplastic polyurethane, allow the grippers to detect and respond to pressure changes, enabling precision control over the objects they manipulate [34]. This ability to integrate various functional materials and sensors in one automated process enhances the grippers' adaptability and reduces production time and costs. The customization capabilities offered by 3D printing allow for the development of grippers tailored to specific tasks, making them particularly valuable in industries like automation, manufacturing, and even apparel, where delicate, flexible, or irregularly shaped items must be handled carefully [35], [36]. Overall, 3D printing opens new possibilities for creating smart, versatile, and multi-functional robotic grippers, advancing the field of soft robotics and industrial automation.

### 5.3 Final remarks

The authors could gratify the outset research queries through the extensive process described in this work. The rapid development of gripper technologies is observable on multiple fronts, many of which apply to stiff and porous fabric materials. Multiple types of pneumatic methods, intrusive mechanisms, adhesive like substances, or atypical electro-adhesion are all actively researched techniques that can be implemented. They directly answer the first research question (RQ1), along with hybrid contraptions. Hybrid technologies are one of the methods used to overcome the limitations of separate mechanisms, along with the use of closed-loop systems and simulations, which relate to the second query (RQ2) of optimisation methods for gripping systems. Sensors can be used to measure both internal and external parameters, which yields a better control system, as was inquired in the third question (RQ3). The data is often used to improve predictive models, which are an important tool. Simulations can be considered to optimise and study various factors and improve the operational efficiency of a system. This answers the last research question (RQ5), as many methods exist to set it up for a particular implementation.

Lastly, each technology has flaws that must not be overlooked. This was explored in detail in the previous section, thus addressing (RQ4) with mitigation strategies relevant to each type of gripping system. Various robot gripper technologies for handling stiff and porous fabrics present distinct limitations and varying degrees of feasibility. Vacuum-based grippers face issues with porous materials due to pressure loss, though strategies like distributed channels and real-time sensors improve efficiency, but they struggle with rough textures and unintentional multi-object picking. Pneumatic mechanisms like Bernoulli-effect grippers provide contactless handling but lack reliability and orientation control, while electro-adhesion offers a strong grip for fibrous materials but is sensitive to surface texture and uneven adhesive forces. Intrusive mechanisms (needles, brushes) provide strong grip and ply separation but risk damaging surfaces, mitigated slightly by thin needles or soft grippers. Adhesion-based technologies, such as dry and active adhesion, offer residue-free alternatives but have challenges generating normal forces, while cryogenic grippers, though strong, risk contamination and need flat surfaces. Soft grippers are adaptable but suffer from low precision and grasping force, and hybrid technologies combine approaches to improve performance but increase system complexity and control challenges. Each technology requires trade-offs between grip strength, material adaptability, surface damage, and complexity.

## 6 Conclusion

The study successfully identified the most recent and relevant research on robot gripper mechanisms. These developments are deemed most suitable for non-woven and compressed polymer fabric materials, characterised by their porosity, rough texture, and shape retention. Three-dimensional shapes made from this material are often used as mould inserts and are currently handled manually. Some of the previous literature review works were analysed to automate that process, and the absence of articles that target porous and simultaneously rigid objects was found. This gap was bridged to some extent and was accomplished by following the PRISMA review structure. Some of the cutting-edge technologies include time-tested methods like vacuum and intrusive needles, though both of them have witnessed significant upgrades to overcome their limitations. For vacuum, it could be in the form of low-pressure high, flow rate designs; for needle grippers, it can be miniaturised needles. Other pneumatic techniques, such as the Bernoulli and the Coanda effects, have proven to be more efficient than vacuums in specific scenarios. New mechanisms like electro-adhesion, switchable adhesion, dry adhesion, soft robotic grippers, and cryogenic grippers are taking hold as well. Lastly, further optimisations can be achieved by means of predictive simulations, sensors integrated within the gripper, and/or by using multiple mechanisms in conjunction. Overall, there is an arsenal of considerable technologies that can be used to design and optimise a robot gripping system for the target material.

The future prospects of this research lie in expanding the exploration of adaptive gripper technologies for stiff and porous fabrics, focusing on refining sensing systems, enhancing material adaptability, and integrating hybrid approaches. Further testing and experimentation with various fabric types in real-world conditions will be essential to validate the effectiveness of these systems. Additionally, incorporating advancements in smart materials and real-time optimization methods will push the boundaries of current knowledge, positioning this research to inform next-generation robotic gripper designs for industrial applications. Collaborative efforts between robotics and material science will be crucial for driving future innovations.

## Funding

This work has been supported by the European Union under the Next Generation EU through a grant of the Portuguese Republic's Recovery and Resilience Plan (RRP) Partnership Agreement, within the scope of the project PRODUTECH R3 - “Agenda Mobilizadora da Fileira das Tecnologias de Produção para a Reindustrialização.”, under the project C645808870-00000067.

This work is also funded by FCT/MEC through national funds and co-funded by the FEDER-PT2020 partnership agreement under the project **UIDB/00308/2020** (DOI 10.54499/UIDB/00308/2020).

## CRediT authorship contribution statement

**Vivek Kumar:** Writing – review & editing, Writing – original draft, Validation, Investigation, Formal analysis, Conceptualization. **Paulo Jorge Coelho:** Writing – review & editing, Writing – original draft, Validation, Investigation, Funding acquisition, Formal analysis, Conceptualization. **Carlos Neves:** Writing – review & editing, Writing – original draft, Validation, Investigation, Funding acquisition, Formal analysis, Conceptualization.

## Declaration of Competing Interest

The authors declare that they have no known competing financial interests or personal relationships that could have appeared to influence the work reported in this paper.

## Figures and Tables

**Table 5:** PRISMA checklist.

Item	Description	Section / Subsection
**Title**	Identify the report as a systematic review, meta-analysis, or both.	Title page 1
**Objectives**	The research question including components such as technology, applications, limitations, and outcomes.	Section 3.1
**Methods**		
Information sources	Key databases searched and search dates.	Section 3.2
Eligibility criteria	Study and report characteristics used as criteria for inclusion.	Section 3.3
Strengths and Limitations of evidence	Brief summary of strengths and limitations of evidence	Section 3
**Results**		
Included studies	Number and type of included studies and participants and relevant characteristics of studies.	Section 4
Synthesis of results	Results for main outcomes	Section 4
**Interpretation and discussion**	General interpretation of the results and important implications.	Section 5
**Source of funding**	Funding and bias declaration and acknowledgments.	Funding

## Appendix A

PRISMA checklist used during synthesis of this work is presented in Table 5.
